# HOX Protein Activity Regulation by Cellular Localization

**DOI:** 10.3390/jdb9040056

**Published:** 2021-12-07

**Authors:** Laure Bridoux, Françoise Gofflot, René Rezsohazy

**Affiliations:** Louvain Institute of Biomolecular Science and Technology, UCLouvain, B-1348 Louvain-la-Neuve, Belgium; laure.bridoux@uclouvain.be (L.B.); francoise.gofflot@uclouvain.be (F.G.)

**Keywords:** HOX proteins, cellular localization, karyopherins, activity regulation

## Abstract

While the functions of *HOX* genes have been and remain extensively studied in distinct model organisms from flies to mice, the molecular biology of HOX proteins remains poorly documented. In particular, the mechanisms involved in regulating the activity of HOX proteins have been poorly investigated. Nonetheless, based on data available from other well-characterized transcription factors, it can be assumed that HOX protein activity must be finely tuned in a cell-type-specific manner and in response to defined environmental cues. Indeed, records in protein–protein interaction databases or entries in post-translational modification registries clearly support that HOX proteins are the targets of multiple layers of regulation at the protein level. In this context, we review here what has been reported and what can be inferred about how the activities of HOX proteins are regulated by their intracellular distribution.

## 1. Introduction

HOX proteins define a family of transcription factors (TFs) initially identified to be crucial actors in controlling the fate of embryonic territories during animal development [1]. They contribute to patterning the body plan of all bilaterian embryos and data support that they also participate in the spatial organization of diploblastic animals such as cnidarians [2,3,4,5]. The functions of HOX proteins, as well as their sequence and structural organization, are well-conserved during evolution. From sequence and genome comparisons, it has been hypothesized that the urbilaterian ancestor of all bilaterian clades had seven *Hox* genes, organized in a single chromosomal cluster [2]. This clustering of *Hox* genes has been maintained in most bilaterian phyla, although some clades underwent a dismantling of their *Hox* clusters, as observed in the nematode *Caenorhabditis elegans* or in ascidians [6,7]. In vertebrate evolution, whole genome duplications gave rise to four paralogous *Hox* clusters (as in mammals) and up to thirteen clusters in fishes [8]. Along these clusters, *Hox* genes can been classified according to paralogy relationships, which reflect both their sequence conservation and relative positioning. Thirteen to fourteen paralogue groups (PG) can thus be recognized in vertebrates [8]. Most significantly, in the context of embryo patterning, the expression pattern of *Hox* genes is collinear with their relative location in the clusters. This implies a spatial collinearity in *Drosophila*, where genes are referred to as “anterior”, “central” or “posterior” with respect to the body segments they control [1,2]. In vertebrates, this implies a spatiotemporal collinearity [9], with the so-called anterior PG (PG 1 to 4) genes being expressed earlier and displaying more rostral boundaries of expression than central (PG 5 to 8) or posterior PG (PG 9 to 13) *Hox* genes.

In addition to their critical involvement in patterning the animal body plan, studies about the functions of HOX proteins revealed that they also participate in organ development, cell differentiation or cell stemness regulation. Furthermore, they maintain activities in adulthood as exemplified in hematopoiesis, neuronal circuit establishment and refinement, or tissue regeneration [10,11,12,13,14]. Consistently with their cellular activities in controlling stemness, differentiation and cell death or proliferation, HOX proteins have been associated with a broad spectrum of pathologies following their mutation or misregulation. These involve pathologies of developmental origin, such as neurological disorders and cardiac malformations, but also include cell pathologies such as cancers [14,15,16].

The roles of HOX proteins have been and remain extensively investigated in a wide range of animal species. Their activities in the context of cancers have also been addressed to a large extent. Far less documented are the modalities of HOX protein action and activity regulation [1].

HOX proteins are TFs that interact with *cis*-regulatory DNA sequences via a conserved homeodomain composed of three alpha-helices. Helix 3 fits into the major groove of the DNA to establish specific contacts with DNA bases, while Helices 1 and 2 are antiparallel and almost perpendicular to Helix 3. The N-terminus of the homeodomain is unstructured, allowing for DNA base contacts via the minor groove. The extreme conservation of Helix 3 and the overall conservation of the homeodomain confer similar DNA recognition properties to HOX proteins. This conservation contrasts with the general functional specificity of HOX proteins [1,17,18,19]. Indeed, while some generic functions have been identified as being shared by all or multiple HOX proteins [20,21], this does not appear to be the general rule. Thus, what confers the functional specificity of HOX proteins is believed to reside in the diversity of protein–protein interactions, which they establish with partner proteins or in the processes regulating their activity in a cell-type specific manner [1,18,19]. 

Few protein–protein interactions involving HOX proteins have been studied in depth. Well-characterized interactions appear to be limited to involve the Three-Amino acid Loop Extension (TALE) family of homeodomain proteins, comprising PBX (Extradenticle, Exd, in *Drosophila*; Ceh-20/60 in *C. elegans*) and MEIS/PREP in vertebrates (Homothorax, Hth, in *Drosophila*; Unc-62 in *C. elegans*). These proteins contribute to extending or to revealing latent DNA-binding specificity to HOX proteins [22]. PBX and MEIS are rather generic TFs and they interact with most HOX proteins. Therefore, the HOX–TALE interaction only partially resolves the paradox of HOX functional specificity [18].

Compared to the TALE proteins, very few HOX–protein interactions have been studied in molecular and functional terms. Proteome-wide interactomic searches involving *Drosophila* or human HOX proteins revealed a wide repertoire of interactions. HOX proteins were shown to interact with TFs but also, significantly, with proteins that are not known to be related to gene regulation. This includes proteins linked to distinct cellular processes such as cell-trafficking, post-translational modification (PTM) of proteins, signal transduction, DNA repair, cell cycling, among many others [23,24,25,26]. Whether these interactions are relevant to the transcriptional activities of HOX proteins, to the regulation of HOX protein activity or to non-transcriptional activities of HOX proteins remains largely unaddressed. However, in some cases, HOX proteins have been demonstrated to play non-transcriptional roles such as in cell signaling, regulation of mRNA translation or DNA repair [27].

Activity regulation of TFs has been extensively studied for some proteins such as the genome guardian p53 [28], MYC [29] or NF-κB [30], for example. Activity regulation often implies PTMs such as phosphorylations, acetylations, ubiquitinations, and protein degradation or stabilization. Although HOX proteins have been demonstrated to be post-translationally modified, the context and consequences of these PTMs remain mostly unknown [31]. Activity regulation of TFs also often implies their reallocation to distinct subcellular compartments, involving nuclear entry or exit as well as their recruitment into organelles, where they can exert non-transcriptional activities. Typically, this is the case for p53, which acts as transcriptional regulator in the nucleus but can be recruited to the mitochondrion where it stimulates the intrinsic apoptotic pathway [32].

How HOX protein activity can be modulated has been scarcely addressed. While recent reviews have focused on the PTMs of HOX proteins, here, we will survey fragmentary data about HOX protein intracellular redistribution, its control and its consequences.

## 2. HOX Protein Localization: Cell-Type and Stage-Specific Changes

The impact of the intracellular redistribution of HOX proteins in cellular and molecular terms has not yet been addressed in vivo. Nonetheless, it clearly appears that HOX protein localization is finely regulated, since it characterizes specific cell types or specific stages along differentiation lineages. For example, HOXA7 displays a dynamic intracellular redistribution during human ovarian folliculogenesis. Granulosa cells that surround the maturing oocyte are devoid of HOXA7 in the most immature follicles. Next, while follicles proceed to the primary stage of maturation, *HOXA7* is switched on and granulosa cells display HOXA7-positive nuclei. Later, as the follicles continue to mature, the subcellular localization of HOXA7 becomes predominantly cytoplasmic [33]. This dynamic supports the concept that HOXA7 expression correlates with granulosa cell proliferation from primordial to primary follicles, with HOXA7 expression being highest in mitotic cells. While the change in intracellular distribution marks the transition from proliferative to secretory granulosa cells, this possibly indicates a change in HOXA7 function in regard to differentiation. During proliferation, HOXA7 could regulate the transition between cell-cycle phases to promote mitosis. One factor modulating the expression of HOXA7 in granulosa cells has been identified to be the oocyte-derived TGFβ superfamily paracrine factor GDF-9 [33].

A dynamic intracellular distribution of HOXB9 has also been highlighted during mammalian oocyte maturation and early embryogenesis [34]. HOXB9 proteins were detected in both mouse and bovine species as early as the immature oocyte stage. From oogenesis to the embryonic blastocyst stage, HOXB9 was identified to be predominantly nuclear. As the first differentiation takes place during compaction of morula-stage embryos and in the blastocyst, HOXB9 expression remains high in trophoblast cells, which will later contribute to the placenta. In contrast, expression decreases and becomes heterogeneous in the inner cell mass, which will give rise to the embryo itself. Next, the inner cell mass will further segregate into the epiblast, which is the precursor of the future embryo, and the primitive endoderm, which contributes to extra-embryonic cells. Although no clear correlation could be drawn between HOXB9 expression and differentiation into epiblast and primitive endoderm, epiblast cells in the bovine species clearly displayed a more homogenous distribution of HOXB9 between the nucleus and cytoplasm. Most significantly, while the primitive endoderm begins to be regionalized in peri-gastrulating mouse embryos, HOXB9 adopts a clear and specific intracellular distribution. It is associated with apical vacuoles in the visceral endoderm, it appears more uniformly cytoplasmic in anterior visceral endoderm, and it is associated with vesicles in the trophectoderm [34]. Although the biological meaning of this dynamic HOXB9 protein distribution remains to be identified, the highly specific intracellular location of HOXB9 in some differentiating cells clearly supports distinct cell-type and stage-specific functions or regulations for HOXB9.

HOXB4, B6 and B13 expression has been associated with epidermis development. Strikingly, the intracellular distribution of these HOX proteins display dynamic changes according to the developmental stages and differentiation steps in humans. HOXB6 was first identified as being present in the supra-basal layer of early developing skin and in the upper layers of late fetal and adult skin. It was detected to be cytoplasmic throughout fetal epidermal development but essentially nuclear in adult skin [35]. HOXB4 was detected in the basal cell layer of the developing epidermis and in the bulge region of the hair follicle, while HOXB4 expression is predominantly supra-basal in the adult epidermis. Here, again, the intracellular distribution of the protein appeared to be dynamic in time and in cell type. It is predominantly cytoplasmic in early fetal stages in the basal cell layer, yet at later stages, it becomes mainly nuclear. In intermediate cells, it shows both cytoplasmic and nuclear distribution at all stages. In the upper cell layers, it is firstly cytoplasmic but becomes both cytoplasmic and nuclear at later fetal and adult stages [36]. Finally, HOXB13 was detected to be cytoplasmic throughout fetal skin development [37]. 

A critical issue that was raised in the study on HOXB6 intracellular distribution during skin development concerns the possible expression/presence of distinct isoforms of the protein [35]. Indeed, it has been reported for several *HOX* genes that alternative mRNAs can be expressed, giving rise to distinct protein isoforms. Most significantly, some alternate transcripts result in the translation of proteins lacking the homeodomain [35,38]. The functions of alternative and truncated HOX proteins have barely been addressed. It has been proposed that truncated proteins may exert a regulatory role with respect to the full-length isoform [39], but the shorter isoforms might fulfill specific functions. It is noteworthy that the nuclear HOXB6 signal has been attributed to the full-length protein, while the cytoplasmic location appeared to relate to the truncated, homeodomain-less HOXB6 variant. In fact, Kömüves et al. [35] reported that an unspliced transcript leading to a shorter HOXB6 was expressed earlier, in undifferentiated cells. The spliced transcript encoding the full-length protein with a homeodomain was induced later upon cell differentiation [35]. Thus, the HOXB6 protein lacking the homeodomain would account for the majority of the cytoplasmic signal detected through the fetal stages of skin development. This additional layer of complexity related to the presence of various isoforms of HOX proteins is underestimated in the issue of the functional specificity of HOX proteins. In fact, HOX protein isoforms could account for intracellular-specific locations, while also establishing distinct protein–protein interactions and displaying particular molecular activities.

## 3. Entering and Leaving the Nucleus

### 3.1. NLS, Predictions and Validations

As expected from their roles as TFs, the nuclear localization of HOX proteins was observed and validated as early as the 1980s [40,41,42]. Gaining access to the nucleus from the cytosol or leaving the nucleus requires passing through nuclear pores, which is enabled by members of the karyopherin family of nuclear transport receptors [43]. Karyopherins facilitate the translocation of proteins by recognizing peptide signals in cargoes: the nuclear localization signals (NLS) for importins and nuclear export signals (NES) for exportins. Canonical NLSs, also called classical or cNLSs, are monopartite or bipartite peptide motifs characterized by basic amino acid residue repeats. DNA binding domains interacting with the ribose-phosphate backbone of the DNA strands often display basic amino acid stretches. Homeodomains in particular harbor basic amino acid clusters close to their N-terminus and in their third α-helix. While these amino acid residues are involved in HOX protein interactions with DNA, these clusters have also been identified or predicted to act as NLSs [43].

**Figure 1:**
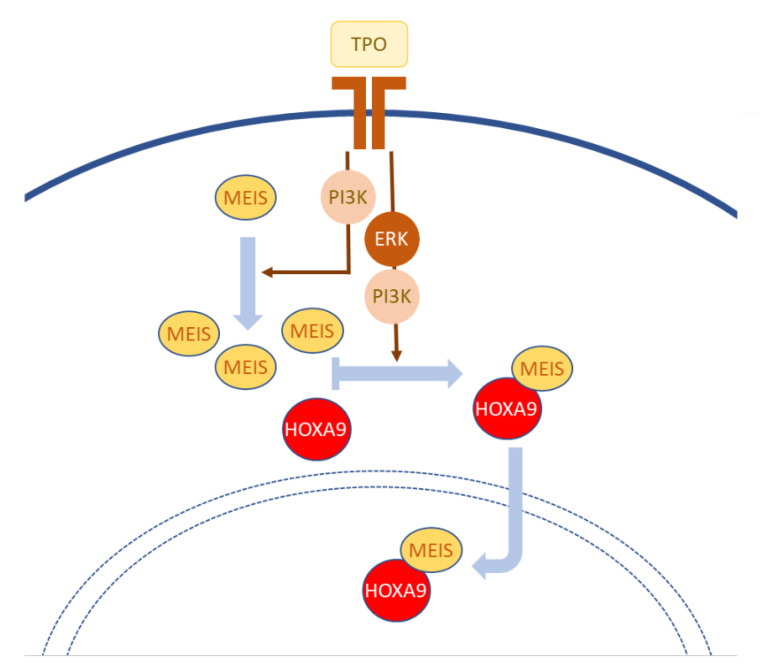
The nuclear translocation of HOXA9 in hematopoietic cells is stimulated by Thrombopoietin (TPO) and relies on its interaction with MEIS. TPO stimulates the expression of MEIS and abundance of MEIS protein, and also promotes the HOXA9–MEIS interaction and the nuclear localization of the HOXA9–MEIS complex. The enhancement of MEIS expression mediated by TPO relies on PI3K signaling. The formation of the HOXA9–MEIS complex and its nuclear entry involves both ERK and PI3K activities (see text for details) [44].

Experimentally, Kirito and colleagues [44] investigated the thrombopoietin (TPO)-mediated induction of HOXA9 nuclear internalization in immature hematopoietic cells. They validated that the basic amino acid clusters at the N-terminus and in the third α-helix of the HOXA9 homeodomain act as NLSs. Interestingly, both NLSs appeared to be required for complete nuclear localization of the protein, since mutating either NLS blunted the nuclear localization of HOXA9. Consistently, a HOXA9 deletion mutant protein devoid of homeodomain displayed a near-exclusive cytoplasmic localization. Interestingly, TPO also stimulated the molecular interaction between HOXA9 and the TALE protein MEIS1 (Figure 1). Mutating the MEIS interaction motif of HOXA9 resulted in an equal nuclear and cytoplasmic distribution of the protein. While dissecting the molecular events elicited downstream of TPO, the authors identified that inhibiting the MEK/ERK pathway disrupted the HOXA9–MEIS1 interaction and HOXA9 nuclear localization. Inhibition of PI3K decreased the levels of MEIS1 mRNA and protein and also affected nuclear accumulation of HOXA9, although in a delayed manner compared to the rapid effect of MEK inhibition. Taken together, these data support a multilayered control of HOXA9 nuclear localization involving two homeodomain-located NLSs and the interaction with MEIS1, which in turn is affected by the activity of kinases [44] (Figure 1). Whether MEIS1 or HOXA9 need to be post-translationally modified and/or if additional interactors are involved in this HOXA9 relocation in response to TPO still needs to be investigated. While upon TPO starvation, the majority of HOXA9 protein can be detected in the cytoplasm, the determinants and mechanisms of HOXA9 nuclear export or cytoplasmic retention also await further study.

In line with identification of an NLS activity associated with the homeodomain, some studies reported the nuclear distribution of truncated HOX proteins consisting of the isolated homeodomain or of the homeodomain plus short N- and C-terminal flanking sequences [45,46,47]. Conversely, proteins lacking the homeodomain display a cytoplasmic localization [44,45]. Experimental verifications of peptide motifs endorsing the role of NLS in HOX proteins remain very rare. Nonetheless, NLS predictions in homeodomain proteins are quite robust, in particular with respect to the two basic amino acid clusters conserved in a vast repertoire of homeodomains. The implementation of bioinformatics tools such as “NLS mapper” to predict the occurrence of classical NLS (http://nls-mapper.iab.keio.ac.jp/cgi-bin/NLS_Mapper_form.cgi (accessed on 19 October 2021)) [48] identified NLS with a high score for all HOX proteins of PG 8 to 13, corresponding to the so-called posterior HOX proteins in *Drosophila,* while PG 1 to 7 proteins usually display NLSs with lower scores (Table 1). In most cases, but not all, these predicted NLSs fit with the basic amino acid clusters of the homeodomain. In three proteins (HOXA5, B5, C9), high score NLSs were predicted to overlap with the short hexapeptide motif shared by most HOX proteins and known to interact with the TALE protein PBX. Similarly, for proteins without a high predicted NLS score, sequences corresponding to or overlapping with this hexapeptide could be revealed by simply lowering the threshold (Table 1). When applying the bioinformatics tool to *Drosophila* proteins, the best prediction identifies the homeodomain of AbdB, which is consistent with the scores obtained for the posterior PGs of human proteins (Table 2). NLSs have also been predicted in sequences overlapping with the Exd-interacting hexapeptide. Finally, the best scores for NLS predictions identify sequences outside the homeodomain in pb and Dfd (Table 2). 

### 3.2. Leaving the Nucleus

**Figure 2:**
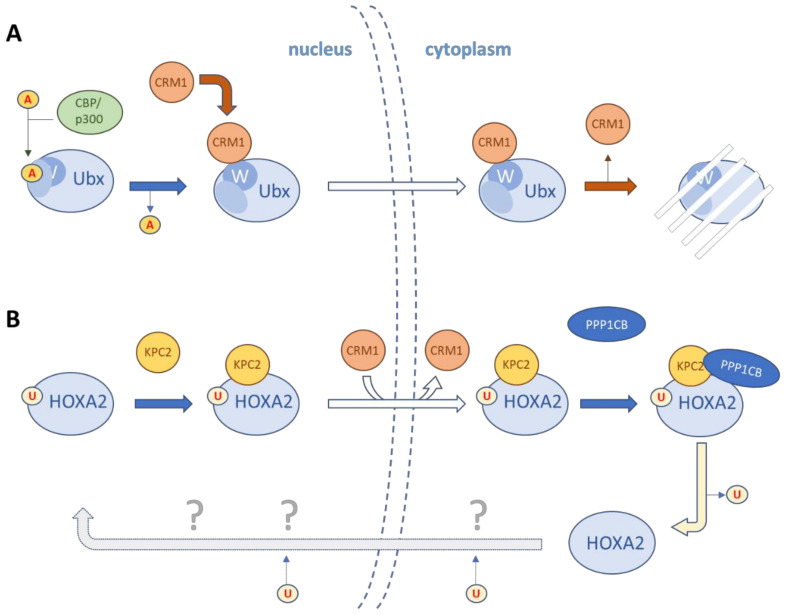
The nuclear exit of Ubx and HOXA2 proteins is a regulated process. (**A**) The nuclear exit of Ubx relies on its interaction with the CRM1/Embargoed exportin involving the PBX/Exd interaction motif, a hexapeptide characterized by an extremely conserved Tryptophan residue (“W”). Accessibility of this hexapeptide is a regulated process involving the acetylation/deacetylation of Ubx. The acetyl-transferase CBP/p300 contributes to this regulatory protein modification. Once in the cytoplasm, Ubx is degraded (see text for details) [49]. (**B**) Model for the activity regulation of HOXA2. HOXA2 interacts with KPC2 in the nucleus, which stimulates its CRM1-dependent nuclear exit. In the cytoplasm, the HOXA2–KPC2 complex is recognized by PPP1CB, which, in turn, promotes the de-ubiquitination of HOXA2. This would establish a ready-to-use supply of HOXA2, which can be primed for nuclear re-entry and transcriptional activity (see text for details) [53].

Removing a TF from the nucleus is a rapid and reversible way for a cell to alleviate its transcriptional input. This could correspond to a fast response from an extracellular stimulus or to a stimulation cessation, as mentioned above with the cytoplasmic relocation or retention of HOXA9 upon TPO depletion. This quite plausible scenario of activity regulation by intracellular relocation suffers again from a lack of experimental evidence as well as from the paucity of data to map extant NES motifs in HOX proteins. One recent elegant study in *Drosophila* identified an unconventional NES in the HOX protein Ubx, which overlaps with the Exd-interacting hexapeptide motif [49]. Ubx and other *Drosophila* HOX proteins share the generic ability to repress autophagy in the fat body of the feeding fly larvae. This repression needs to be lifted once larvae start entering metamorphosis, which requires active autophagy. The regulatory scenario proposed by the authors is that in wandering larvae, before the onset of metamorphosis, an active clearance of Ubx proteins from the nucleus followed by its rapid degradation allows autophagy to be de-repressed (Figure 2A). Sequence deletions, NES predictions and point mutations identified an unconventional NES overlapping with the hexapeptide. Both the NES and the integrity of the hexapeptide were reported to be required for the interaction of Ubx with the major *Drosophila* exportin Embargoed (the homologue of the vertebrate karyopherin CRM1) [49]. Embargoed and NES/hexapeptide-dependent nuclear export of Ubx were further reported to be shared by other *Drosophila* HOX proteins (Scr and Dfd) as well as to be conserved in the mammalian orthologue HOXA5. Mechanistically, the switch from the nuclear residence of Ubx and its rapid removal from the nucleus was hypothesized to rely on NES unmasking. Classically, masking or unmasking small peptide motifs such as NLS or NES imply protein–protein interactions, as exemplified for the PBX/Exd nuclear entry, which relies on MEIS/PREP/Hth interaction [50,51,52]. Duffraisse et al. [49] suggested that the masking of the Ubx NES could involve protein acetylation, since inhibiting the acetyl transferase CBP/p300 induced the nuclear exit of Ubx, and concurrently induced premature autophagy de-repression. In contrast, NES-mutated Ubx remained refractory to the depletion of CBP/p300 by RNAi [49] (Figure 2A).

The regulation of HOX protein activity by export from the nucleus also received support from a study about the HOXA2 interactors KPC2 and PPP1CB. KPC2 is a subunit of the Kip1 ubiquitination promoting complex (KPC) and PPP1CB is the beta catalytic subunit of the serine/threonine protein phosphatase PP1 (Figure 2B). Both KPC2 and PPP1CB were identified as interactors of HOXA2 in a proteome-wide yeast two-hybrid screening [45,53]. As a subunit of a ubiquitin ligase complex, KPC2 was assumed to negatively regulate the activity of HOXA2 by promoting its degradation, similar to the KPC2 target protein p27^kip1^ [54]. However, KPC2 appeared to decrease the activity of HOXA2 by stimulating its cytoplasmic relocation without impacting its degradation. Inhibition of CRM1 exportin appeared to trap the HOXA2–KPC2 interaction in the nucleus, leading to the proposed model that KPC2 primarily interacts with HOXA2 in the nucleus and causes its translocation towards the cytoplasm [45]. PPP1CB has been demonstrated to modulate the activity of the homeodomain protein NKX2.5, while promoting its perinuclear redistribution [55]. A similar regulatory redistribution appeared to operate for HOXA2. In addition, the PPP1CB–HOXA2 interaction pattern in the cell appeared to overlap with that of HOXA2–KPC2. Deneyer et al. [53] provided evidence that KPC2 and PPP1CB are also able to interact with each other. The model supported by the dissection of these interactions is that KPC2 primarily interacts with HOXA2 to promote its translocation towards the cytoplasm and that cytoplasmic PPP1CB either enhances this translocation or favors the cytoplasmic retention of HOXA2. Next, PPP1CB and KPC2 contribute to decrease the relative abundance of ubiquitinated HOXA2, and KPC2 also increases HOXA2 half-life. Together, KPC2 and PPP1CB contribute to regulate the activity of HOXA2 while promoting both its cytoplasmic location and stability [45,53]. These interactors would therefore allow the maintenance of a ready-to-use cytoplasmic store of HOXA2 (Figure 2B). Whether HOXA2 de-ubiquitination contributes to its stabilization or cytoplasmic localization—or both—needs to be further investigated. Additionally, one attractive possibility would be that ubiquitination might define a signal promoting HOXA2 nuclear entry or residence. The capacity of KPC2 to interact with HOX proteins was further confirmed for proteins of anterior, central and posterior PGs [45]. The intracellular pattern of KPC2–HOX interaction, however, appeared rather diverse, from mainly nuclear to cytoplasmic. This underlines that while the interaction with KPC2 might be generic to HOX proteins, the outcome of this interaction and the regulatory influence of KPC2 on distinct HOX activities might be specific.

In this complex interplay between HOXA2, KPC2 and PPP1CB, the CRM1 exportin seems to be involved. The peptide motif(s) at work as NES, however, have not been identified. According to Fung et al. [56], NES can be classified due to distinct patterns of hydrophobic amino acid-rich motifs, with four core hydrophobic amino acids separated by one to three other amino acid residues. Online tools are available to search for candidate NES sequences such as NetNES [57] or LocNES [58]. Searching for peptide motifs that respect NES consensus sequences or scanning sequences with NetNES or LocNES did not result in consistent predictions for HOX proteins (not shown). However, the first helix of the homeodomain is predicted to possess NES activity in some homeoproteins, mainly corresponding to some anterior (PG 1–4) and posterior (PG 9–10) PG proteins. If using HOXA5 as an input, NetNES1.1 identifies a candidate NES at the end of the second helix of the homeodomain, which does not correspond to what has been experimentally identified by Duffraisse et al. [49]. In contrast, searching for sequences matching the NES consensus sequence Φ-X2/3-Φ-X2/3-Φ-X-Φ (with Φ being L, V, I, F or M), the hexapeptide sequence of PG4/Dfd and PG5/Scr proteins is identified and seems to be evolutionary conserved in most bilaterian sequences (chordates, nematode *C. elegans* and *Drosophila*). Using this consensus sequence also identifies the first helix of the homeodomain of lab and pb in *Drosophila*, which is consistent with what has been predicted for mammalian PG 1 and 2 proteins. It also identifies the third helix of the Ubx homeodomain, which again does not correspond to the functional data reported [49]. All this highlights the looseness in predictions and the necessity to functionally validate NESs in the context of HOX protein activity regulation.

### 3.3. Leaving the Nucleus to Leave Cells?

As with the NLS motifs, looking at other closely related homeodomain proteins might provide insight into NES and their roles in regulating HOX protein functions. Of particular interest are the studies of Prochiantz and collaborators [47,59,60], which identified that homeodomain sequences, and in particular the third helix, display cell-permeable properties. If physiologically relevant, this property implies that some homeodomain proteins could be extracellular and possess paracrine functions. This has been functionally validated for OTX, PAX and En proteins [61,62,63]. Gaining an extracellular function necessitates proteins to be released by cells. Most significantly, an NES sequence was identified as extending between helices 2 and 3 of the En homeodomain, which appeared to be required for its unconventional secretion [64,65,66]. Consequently, the extracellular release of En would require its transit through the nucleus. This phenomenon appears to be regulated by PTM adducts [67]. With a canonical third helix in their homeodomain, HOX proteins share the capacity to enter cells in a receptor- and energy-free manner [47,59,60], and a possible paracrine activity for HOX proteins has also been hypothesized [68,69].

### 3.4. Interactions with Karyopherins and Proteins Associated with Cytoplasmic Organelles

To obtain insight into possible activity regulations involving the intracellular redistribution of HOX proteins, searching protein–protein interaction databases might be informative. Some *Drosophila* and human HOX proteins have been involved in wide interactome screenings, although some studies limited the search to TFs or nuclear gene regulators [23,24,25,26]. In addition, mapping the overall human interactome also identified putative HOX interactors [70,71]. These interactions are compiled in publicly available databases such as the BioGrid (https://thebiogrid.org/ (accessed on 7 December 2021)) or the IntAct Molecular Interaction Database (https://www.ebi.ac.uk/intact/home (accessed on 7 December 2021)), and their entries largely overlap. They can collectively be accessed and interrogated due to the PSICQUIC initiative (http://www.ebi.ac.uk/Tools/webservices/psicquic/view/main.xhtml (accessed on 7 December 2021)) [72].

Intriguingly, very few entries identified candidate interactions with karyopherins. Only the *Drosophila* TNPO transportin (Karyopherin-β2) is reported to interact with Ubx [24]. No karyopherin is identified in databases as interactors of HOX proteins in mammalian proteomes and only one entry for HOXA1 identified an RAN-binding protein involved in nuclear pore translocation (namely RANBP3). The paucity of database entries connecting HOX proteins and karyopherins or the nuclear translocation machinery underlines the scarceness of studies addressing the intracellular distribution of HOX proteins. 

In contrast, some interactions identified proteins active in different cytoplasmic compartments or at the plasma membrane, such as focal adhesion molecules, cytoskeletal components, numerous signal transduction molecules (i.e., intracellular receptor binding adapters and kinases), ribosome subunits and vesicular traffic-associated proteins for which functional studies are lacking [23,24]. The functional validation of these candidate interactions and their possible outcomes require investigation. In particular, a crucial issue in that context is to determine if these interactions are relevant to the regulation of the transcriptional activity of HOX proteins by their intracellular relocation. Alternatively, these interactions could concern some cytoplasmic functions of HOX proteins (see next section). Finally, it cannot be ruled out that interactors with known functions in the cytoplasm might also have unknown transcriptional activities, which remain to be unveiled. Of note, karyopherins such as CRM1 have been demonstrated to interact with the chromatin and to contribute to gene regulation [73].

## 4. If Not in the Nucleus, What Do HOX Proteins Do?

As already detailed for Ubx and the HOXA2–KPC2–PPP1CB interaction, relocating TFs to the cytoplasm is a way to regulate their gene regulatory activity, to promote their cytoplasmic degradation (Ubx) or, on the contrary, to keep a ready-to-use mobilizable supply of proteins which can be primed to re-enter the nucleus. A neglected issue, however, concerns the possible non-transcriptional functions of HOX proteins. Experimental evidence indeed supports that HOX proteins are regulators of the cell-cycle and DNA repair. In the cytoplasm, HOX proteins have been involved in the control of mRNA translation or in modulating cell signaling [27]. Some TFs such as ETS family members have been shown to regulate mRNA splicing [74]. This raised the possibility that some TFs exit the nucleus along with target mRNAs, which could also be the case for HOX proteins.

HOXA2 has been reported to stimulate the degradation of the ubiquitin-ligase RCHY1, which, in turn, leads to the stabilization of the genome guardian p53. The HOXA2–RCHY1 interaction, however, seems to take place in the nucleus [75,76]. HOXA1 has also been demonstrated to stimulate the nuclear entry and activity of NF-κB. The HOXA1-mediated translocation of NF-κB, which seems relevant to breast tissue oncogenesis, takes place upstream of the Iκ-B inhibitor and involves cytoplasmic interactions with the signaling modulators TRAF2 and RBCK1 [77]. HOXA1 can interact with numerous other signaling regulators; some interactions display a clear cytoplasmic pattern [23]. However, the functional consequences of these interactions remain to be addressed. As previously mentioned, while these interactions might affect the functions of the interactors, as in the HOXA1/NF-κB functional interaction, they can also be regulatory towards HOX activity. HOXA9, for example, interacts with SMAD4 in the cytoplasm and this interaction modulates the activity of HOXA9. SMAD4 restrains the nuclear entry of HOXA9 to prevent excessive hematopoietic stem and progenitor cell expansion and leukemogenesis [78].

Interactions of HOX proteins with proteins involved in cell signaling, vesicular trafficking, and cell shape regulation, for example, are a recurrent observation when searching interactome databases [23,24,70,71]. Although most of these interactions have not been characterized for their intracellular distribution, among the ones that have been studied further, several have been shown to take place in the cytoplasm [23]. When examining the patterns of protein–protein interactions, with Bimolecular Fluorescence Complementation (BiFC), for example, some interactions seem to be quite specific, displaying diffuse cytoplasmic distribution or punctuated signals (see Figure 3 in [23]). When interaction patterns appear to be discretely distributed, an important and still lacking piece of information remains the identification of the intracellular structures at which interactions take place. Punctuated signals might simply result from artifactual protein aggregation. Alternatively, they might identify organelles such as endosomes or mitochondria. Localizing interactions might be of primary importance to identify new regulatory functions of HOX proteins, such as those involved in endosomal signaling or mitochondrial-linked apoptosis. Although these possibilities are still quite speculative, it is worth keeping them in mind for the future. For these possible non-transcriptional functions, regulating the intracellular redistribution of HOX proteins by protein–protein interactions or PTMs is an issue that will deserve more interest. A major challenge, however, will be to tackle this issue in biological contexts in vivo.

## 5. Not to Conclude…

Like several aspects of the molecular biology of HOX proteins, activity regulation at the post-translational level suffers from a tremendous lack of investigation. This is in sharp contrast with other well-studied TFs such as p53 or NF-κB. One possible explanation is that HOX proteins have primarily been studied in the context of developing embryos and at the organismal level, considering their spectacular functions in defining the fate of territories or in orchestrating organogenesis. In contrast, regulators such as p53 or NF-κB, known primarily for their control of cell-fate decisions (i.e., apoptosis, proliferation, DNA repair, and cytokine secretion), have been more deeply studied in in vitro cellular models. This approach has allowed for the questions regarding protein regulation more amenable than in whole embryos. These biases in study paradigms, justified by what is known in terms of biological activities of proteins, should however not conceal that activity regulation of proteins involved in complex developmental processes might be relevant as well. For example, if in some cell types the stability of a HOX protein is several hours, as observed in vitro for HOXA1 [79], this implies that the protein remains active and thereby can influence the fate of cell lineages that have long since stopped expressing the gene. Characterizing the regulation of HOX protein activity by cellular localization or re-location, either in the course of development or in complex tissue environments, will complete the fragmentary picture we currently have regarding how HOX proteins contribute to shaping animal bodies.

## Figures and Tables

**Table 1:** Best score predictions for NLS sequences of human HOX proteins, generated by cNLS mapper [48]. (HX, hexapeptide; HD, homeodomain; Score: grey >3–5≥; orange >5–7≥; red > 7)).

	HoxA	HoxB	HoxC		HoxD
1	HD	HX + HD			N-ter + HD
2	HD	HD			
3	HD	HD			HD
4	HX	HD	HX		HD
5	HX + HD	HX + HD	HD		
6	HD	HD	HD		
7	HD	HD			
8		HD	HD		HD
9	HD	HD	HX	HD	HD
10	HD		HD		HD
11	HD		HD		HD
12			HD		HD
13	HD	HD	HD		HD

**Table 2:** Best score predictions for NLS sequences of *Drosophila* HOX proteins, generated by cNLS mapper [48]. (HX, hexapeptide; HD, homeodomain; N-ter, N-terminus; C-ter, C-terminus; Score: grey >3–5≥; orange >5–7≥; red > 7)).

lab	HX + HD	
pb	N-ter	
Dfd	HX	C-ter
Scr	HX + HD	
Antp	HD	
Ubx	HD extremities + HD surrounding	
abd-A	HD	
Abd-B	HD	

## Data Availability

Not applicable.

## Abbreviations

NES	Nuclear Export Signal
NLS	Nuclear Localization Signal
PG	paralogue group
PTM	Post-Translational Modification
TALE	Three-Amino Acid Long Extension
